# Laboratory evaluation of the contact irritancy of a clothianidin solo formulation vs. clothianidin-deltamethrin mixture formulations for indoor residual spraying against pyrethroid-resistant *Anopheles gambiae* sensu lato

**DOI:** 10.1186/s13071-024-06265-x

**Published:** 2024-04-10

**Authors:** Thomas Syme, Boris N’dombidjé, Aicha Odjo, Martial Gbegbo, Damien Todjinou, Corine Ngufor

**Affiliations:** 1 London School of Hygiene and Tropical Medicine, London, UK; 2 Centre de Recherche Entomologique de Cotonou, Cotonou, Benin; 3 Pan African Malaria Vector Research Consortium (PAMVERC), Cotonou, Benin

**Keywords:** Indoor residual spraying, Contact irritancy, Vector control, Neonicotinoids, Insecticide resistance

## Abstract

**Background:**

Clothianidin-based indoor residual spraying (IRS) formulations have become available for malaria control as either solo formulations of clothianidin or a mixture of clothianidin with the pyrethroid deltamethrin. While both formulations have been successfully used for malaria control, studies investigating the effect of the pyrethroid in IRS mixtures may help improve our understanding for development of future IRS products. It has been speculated that the irritant effect of the pyrethroid in the mixture formulation may result in shorter mosquito contact times with the treated walls potentially leading to a lower impact.

**Methods:**

We compared contact irritancy expressed as the number of mosquito take-offs from cement surfaces treated with an IRS formulation containing clothianidin alone (SumiShield® 50WG) to clothianidin-deltamethrin mixture IRS formulations against pyrethroid-resistant *Anopheles gambiae* sensu lato under controlled laboratory conditions using a modified version of the World Health Organisation cone bioassay. To control for the pyrethroid, comparison was made with a deltamethrin-only formulation. Both commercial and generic non-commercial mixture formulations of clothianidin and deltamethrin were tested.

**Results:**

The clothianidin solo formulation did not show significant contact irritancy relative to the untreated control (3.5 take-offs vs. 3.1 take-offs, *p* = 0.614) while all deltamethrin-containing IRS induced significant irritant effects. The number of take-offs compared to the clothianidin solo formulation (3.5) was significantly higher with the commercial clothianidin-deltamethrin mixture (6.1, *p* = 0.001), generic clothianidin-deltamethrin mixture (7.0, *p* < 0.001), and deltamethrin-only (8.2, *p* < 0.001) formulations. The commercial clothianidin-deltamethrin mixture induced similar contact irritancy as the generic clothianidin-deltamethrin mixture (6.1 take-offs vs. 7.0 take-offs, *p* = 0.263) and deltamethrin-only IRS (6.1 take-offs vs. 8.2, *p* = 0.071), showing that the irritant effect in the mixture was attributable to its deltamethrin component.

**Conclusions:**

This study provides evidence that the enhanced contact irritancy of the pyrethroid in clothianidin-deltamethrin IRS mixtures can shorten mosquito contact times with treated walls compared to the clothianidin solo formulation. Further trials are needed to directly compare the efficacy of these formulation types under field conditions and establish the impact of this enhanced contact irritancy on the performance of IRS mixture formulations containing pyrethroids.

**Graphical Abstract:**

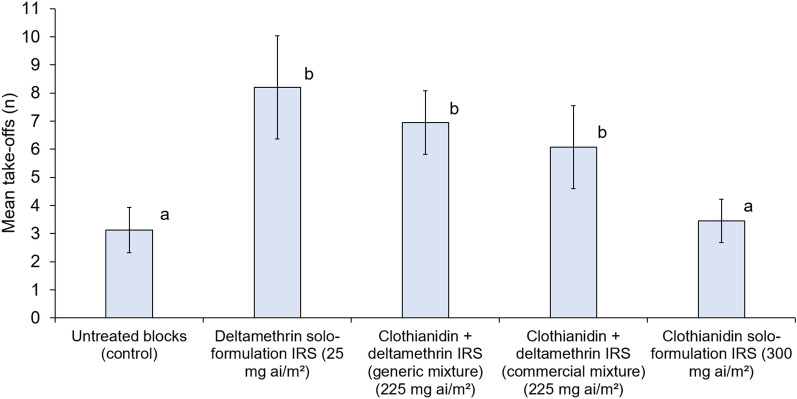

**Supplementary Information:**

The online version contains supplementary material available at 10.1186/s13071-024-06265-x.

## Background

Indoor residual spraying (IRS) is one of the two vector control interventions recommended by the World Health Organisation (WHO) for large-scale deployment in areas with ongoing malaria transmission [1]. Systematic reviews of cluster-randomised controlled trials (cRCTs) and quasi-experimental studies have established the public health value of IRS [2, 3], and it has contributed significantly to reductions in malaria observed in sub-Saharan Africa since 2000 [4]. The formulations widely used for IRS traditionally fell into four insecticide classes: pyrethroids, organochlorines, organophosphates, and carbamates. Unfortunately, resistance to these insecticides is increasing in malaria vectors throughout sub-Saharan Africa [5], threatening to undermine the effectiveness of IRS. New effective, long-lasting insecticides are needed to improve the viability of IRS for malaria control.

The first new insecticide class developed for IRS was the neonicotinoid clothianidin. An IRS formulation containing clothianidin alone (SumiShield® 50WG) initially demonstrated improved and prolonged efficacy against insecticide-resistant malaria vectors in experimental hut trials and in small and large-scale village-randomised trials in Africa [6, 7] and India [8, 9]. Based on this, SumiShield® 50WG was added to the WHO list of prequalified vector control products in 2017 [10], making clothianidin the first new mode of action adulticide to be approved for malaria vector control in over 40 years. In subsequent years, two additional IRS formulations (Fludora® Fusion, 2GARD) containing a mixture of clothianidin and the pyrethroid deltamethrin were prequalified [10] after demonstrating evidence of entomological efficacy [11–13]. IRS with clothianidin has consequently been scaled up in recent years. Clothianidin-based IRS products were used by 10 countries for IRS campaigns in 2020 [14] and accounted for approximately 55% of the total volume of IRS sachets procured in 2022 [15].

While both mixture and solo IRS formulations of clothianidin have been successfully used for malaria control, studies investigating the effect of the pyrethroid in IRS mixtures may help inform decision-making for control programmes and improve our understanding for development of future IRS products. An experimental hut trial in Benin demonstrated a significantly reduced performance of an IRS mixture formulation of chlorfenapyr and alpha-cypermethrin compared to a formulation containing chlorfenapyr alone [16]. It was speculated that this finding may be attributable to the irritant effect of the pyrethroid in the mixture formulation resulting in shorter contact times with the treated walls compared to the solo formulation. Indeed, a more recent experimental hut trial showed higher levels of mosquito exiting and lower mortality with a wettable powder (WP) formulation of a clothianidin-deltamethrin mixture compared to a WG solo formulation of clothianidin applied at the same dose. Further studies are however needed to quantify the excitorepellent effect of the pyrethroid and investigate its potential impact on the efficacy of the mixture IRS formulations compared to the solo formulations. Laboratory studies allow for more precise measurement of the behavioural and physiological responses of mosquitoes to vector control interventions under controlled conditions and thus can be used to quantify and compare the contact irritancy of mixture and solo IRS formulations.

In the present study, we compared the contact irritancy of an IRS formulation containing clothianidin alone (SumiShield® 50WG) to clothianidin-deltamethrin IRS mixtures against pyrethroid-resistant *Anopheles gambiae* under controlled laboratory conditions using a modified version of the WHO cone bioassay. To control for confounding effects of the pyrethroid and formulation type, a deltamethrin-only IRS formulation and both commercial and generic (i.e. non-commercial) mixture formulations of clothianidin and deltamethrin were tested. Susceptibility bioassays were also performed to characterise the resistance status of the laboratory-reared mosquito strains to clothianidin and pyrethroids and support interpretation of the results.

## Methods

This study was performed at CREC/LSHTM bioassay laboratory in Cotonou, Benin, in July 2022.

### Mosquito strain characterisation

Laboratory bioassays were performed with laboratory-reared susceptible and pyrethroid-resistant strains of the major malaria vector *An. gambiae*. Both strains are maintained at CREC/LSHTM insectary in Cotonou, Benin. The species composition and resistance profiles of the susceptible and pyrethroid-resistant strains used for the study are described below.

- *Anopheles gambiae* sensu stricto Kisumu strain is an insecticide-susceptible reference strain originating from Kisumu, western Kenya.
- *Anopheles gambiae* sensu lato Covè strain is an insecticide-resistant field strain that is F1 progeny of mosquitoes collected from CREC/LSHTM field station in Covè, southern Benin. Prior studies show that the strain exhibits a high frequency of resistance to pyrethroids and organochlorines but remains susceptible to other insecticide classes including clothianidin. Resistance is mediated by the knockdown resistance (*kdr*) L1014F mutation and overexpression of P450 enzymes, notably CYP6P3 [17].

### Susceptibility bioassays

Susceptibility bioassays were performed according to WHO guidelines [18] to assess the susceptibility of the *An. gambiae* s.l. Covè strain to the AIs in the IRS treatments. Mosquitoes were exposed in tube tests to filter papers impregnated with the discriminating concentration of deltamethrin (0.05%) and in bottle bioassays to the discriminating concentration of clothianidin (4 µg) to assess susceptibility to these insecticides. Further exposures were performed with 5 × and 10 × the discriminating concentration of deltamethrin to assess pyrethroid resistance intensity. To assess synergism and the role of P450s in pyrethroid resistance, mosquitoes were also exposed to the discriminating concentration of deltamethrin (0.05%) with pre-exposure to the cytochrome P450 monooxygenase (P450) inhibitor piperonyl butoxide (PBO) (4%). Insecticide-treated filter papers used for tube tests were obtained from Universiti Sains Malaysia. To prepare test bottles for clothianidin susceptibility bioassays, predetermined quantities of technical grade clothianidin were dissolved in acetone and Mero® (800 ppm concentration) to obtain a 4 µg/ml stock solution. Test bottles were coated by introducing 1 ml of a pre-prepared stock solution into bottles and rotating them using a tube roller before leaving them to dry for 2 h. A total of 100 mosquitoes aged 3–5 days were exposed to each insecticide and dose for 60 min in four replicates of approximately 25. Parallel exposures were performed with PBO alone, silicone oil + acetone-impregnated papers, and acetone + Mero®-coated bottles as controls. Similar numbers of the susceptible *An. gambiae* s.s. Kisumu strain were exposed to the discriminating concentrations of deltamethrin (0.05%) and clothianidin (4 µg) and appropriate controls to validate the test. Knockdown was recorded at the end of exposure, after which mosquitoes were transferred to untreated containers and provided access to 10% (w/v) glucose solution. Mortality was recorded after 24 h for all exposures. Tests and subsequent mortality recordings were performed at 27 ± 2 °C and 75 ± 10% relative humidity.

### Contact irritancy cone bioassays

The WHO cone bioassay is a standard methodology used to assess the residual efficacy of IRS treatments [19]. A modified version of this test method using video recordings was performed to compare the contact irritancy of a clothianidin solo formulation and clothianidin-deltamethrin mixture formulations for IRS applied to cement blocks under laboratory conditions. The contact irritancy of a clothianidin solo formulation (SumiShield® 50WG, Sumitomo Chemical Co., Ltd.”) was compared to wettable powder (WP) formulations of clothianidin-deltamethrin mixtures. To control for confounding effects of the pyrethroid, comparison was made to a deltamethrin-only water-dispersible granule IRS formulation. Two types of WP clothianidin-deltamethrin mixtures were tested, one commercially available formulation and one non-commercial generic formulation developed for this study. All commercial products were applied at the label application rate. Untreated cement blocks were used as a negative control. The following five treatments were thus compared in contact irritancy bioassays:

i. Untreated blocks (control)
ii. Deltamethrin solo formulation WG IRS applied at 25 mg ai/m^2^
iii. Generic clothianidin-deltamethrin WP IRS applied at 200 mg ai/m^2^ clothianidin and 25 mg ai/m^2^ (225 mg ai/m^2^)
iv. Commercial clothianidin-deltamethrin WP IRS applied at 200 mg ai/m^2^ clothianidin and 25 mg ai/m^2^ (225 mg ai/m^2^)
v. Clothianidin solo formulation WG IRS applied at 300 mg ai/m^2^

#### Preparation and treatment of block substrates

Cement blocks were prepared by filling Petri dishes with a 1:1 mixture of cement and sand and leaving the paste to cure for 30 days. Insecticide solutions at desired concentrations were prepared by mixing IRS formulations with predetermined volumes of water. The blocks were sprayed with insecticide at the application rates using a Potter Spray Tower to ensure a homogeneous and accurate deposition of insecticide on the block substrates. Blocks were weighed before and after spraying to ensure the correct target spray volume was delivered; any blocks falling outside the pre-determined acceptable weight range were discarded. A total of four replicate blocks were prepared per treatment arm.

#### Contact irritancy video cone bioassay procedure

**Fig. 1:**
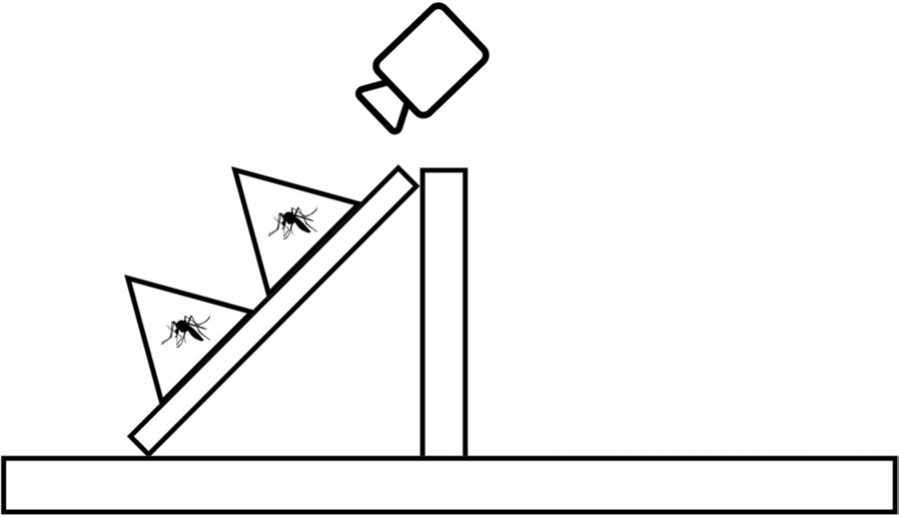
Experimental set-up for contact irritancy cone bioassays with video camera set up in view of the cones

Contact irritancy video cone bioassays were performed 1 week after treatment application with the pyrethroid-resistant *An. gambiae* s.l. Covè strain. Video cameras were set up in view of the cones to record the contact irritancy response of mosquitoes during exposure to block surfaces (Fig. 1 and Additional file 1: Fig. S1). Mosquitoes were introduced individually into each cone, which was plugged with a small sheet of plastic rather than cotton to reduce the surface area of untreated refugia where mosquitoes could rest. Video recordings then began immediately post-introduction and continued for a total of 4 min. To allow time for the mosquito to settle, the first minute of the recording was not considered; hence, contact irritancy observations were made for a total of 3 min. After 4 min, the video recording was stopped and exposure to the IRS-treated block continued until the WHO-recommended 30 min exposure time was reached [19]. At the end of exposure, mosquitoes were transferred to labelled cups and provided access to a 10% (w/v) glucose solution soaked in a piece of cotton. Knockdown was recorded 60 min after exposure and mortality every 24 h up to 120 h. Mortality after 120 h was used as the primary outcome measure for product efficacy to account for the delayed action of clothianidin. Tests were repeated until a total of 10 mosquitoes were exposed to each replicate block corresponding to a total of 40 mosquitoes per treatment arm. Video recordings were then observed separately by two technicians to record the number of times mosquitoes took off following contact with the block surface over 3 min. If there was a discrepancy in the number of take-offs recorded between the two observations, a third technician would observe the video to determine the correct value. Tests and delayed mortality recordings were performed at 27 ± 2 °C and 75 ± 10% relative humidity. Contact irritancy was expressed in terms of the average number of take-offs during the 3-min video recording. Knockdown after 60 min and delayed mortality every 24 h up to 120 h after exposure were also used as secondary outcome measures of product efficacy.

### Data analysis

Differences in contact irritancy between IRS treatments expressed as the number of take-offs were analysed using negative binomial regression in Stata version 17. The model included fixed effects for the replicate blocks and day of testing. Proportional knockdown and mortality were plotted on graphs to visualise differences between treatments for each outcome. Susceptibility bioassay data were interpreted according to WHO criteria [18].

### Ethical considerations

The Animal Welfare Ethics Review Board of the London School of Hygiene & Tropical Medicine issued approval for the use of guinea pigs for mosquito blood-feeding (Ref. 2020-01B). The study did not involve human participants and thus did not require further ethical review.

## Results

### Susceptibility bioassay results

Mortality of the *An. gambiae* s.l. Covè strain was very low (3%) following exposure to the discriminating concentration of deltamethrin (0.05%) demonstrating a high frequency of pyrethroid resistance (Table 1). Mortality was higher with 5× (34%) and 10× (67%) the discriminating concentration but failed to achieve the WHO susceptibility cut-off (≥ 98%) indicating high intensity pyrethroid resistance. PBO pre-exposure improved the mortality response to the discriminating concentration of deltamethrin (41%) but failed to restore full susceptibility suggesting P450s were partially responsible for pyrethroid resistance. PBO alone induced negligible mortality (3%). In contrast, 100% mortality was recorded following exposure to the discriminating dose of clothianidin (4 µg) demonstrating susceptibility of the Covè strain to neonicotinoids. The discriminating concentrations of deltamethrin and clothianidin induced 100% mortality of the *An. gambiae* s.s. Kisumu strain, confirming susceptibility. No mortality (0%) was observed with silicone oil and acetone + Mero® controls with either strain.

**Table 1:** World Health Organisation susceptibility bioassay results with the *Anopheles gambiae* sensu stricto Kisumu strain and *An. gambiae* sensu lato Covè strain

Strain	Treatment	Dose	*N*	*N* dead 24 h	% dead 24 h	95% CIs
Kisumu	Silicone oil (control)	–	100	2	2.0	0.0–4.7
	Deltamethrin	0.05%	100	100	100	–
	Acetone + Mero® (control)	–	100	0	0	–
	Clothianidin	4 µg	100	100	100	–
Covè	Silicone oil (control)	–	100	0	0.0	–
	Piperonyl butoxide	4%	92	3	3.3	0.0–6.9
	Deltamethrin	0.05%	96	3	3.1	0.0–6.6
		0.25%	99	34	34.3	25.0–43.7
		0.5%	93	62	66.7	57.1–76.2
	Piperonyl butoxide + Deltamethrin	4% + 0.05%	93	38	40.9	30.9–50.9
	Acetone + Mero® (control)	–	100	0	0.0	–
	Clothianidin	4 µg	92	92	100	–

Approximately 100 mosquitoes were exposed to each treatment in four replicates of 20–25

### Contact irritancy cone bioassay results

#### Contact irritancy results

**Fig. 2:**
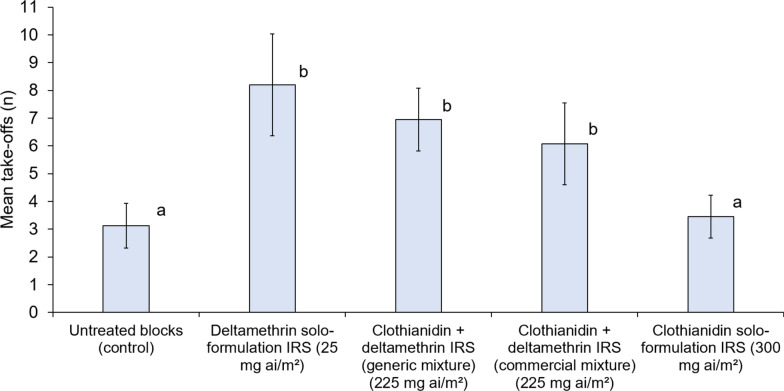
Mean number of take-offs of the pyrethroid-resistant *Anopheles gambiae* sensu lato Covè strain in contact irritancy video cone bioassays. A total of 40 mosquitoes were individually introduced into cones attached to treated cement blocks and filmed for 3 min to record take-offs. Bars bearing the same letter do not differ significantly at the 5% level (i.e. *p* ≥ 0.05) according to regression analysis. Error bars represent 95% confidence intervals

Mosquitoes exposed to untreated control blocks took off on average 3.1 times during the 3-min observation period. The clothianidin solo formulation did not show significant contact irritancy relative to untreated control (3.5 take-offs vs. 3.1 take-offs, *p* = 0.614) (Fig. 2). In contrast, the other IRS treatments containing deltamethrin induced significantly higher contact irritancy than the clothianidin solo formulation and the control. The numbers of take-offs compared to clothianidin solo formulation were significantly higher with the commercial clothianidin-deltamethrin mixture (6.1 vs. 3.5, *p* = 0.001), the generic clothianidin-deltamethrin mixture (7.0 vs. 3.5, *p* < 0.001), and the deltamethrin-only (8.2 vs. 3.5, *p* < 0.001) IRS formulations. The commercial clothianidin-deltamethrin mixture induced similar contact irritancy as the generic clothianidin-deltamethrin mixture (6.1 take-offs vs. 7.0 take-offs, p = 0.263) and deltamethrin-only IRS (6.1 take-offs vs. 8.2, p = 0.071). These comparisons demonstrate that enhanced irritancy of clothianidin-deltamethrin mixtures was primarily attributable to its deltamethrin component rather than formulation differences. Full summary contact irritancy bioassay results (Additional file 1: Table S1) and links to video recording excerpts showing the irritating effects of the commercial clothianidin-deltamethrin mixture compared to the absence of irritating effects with the clothianidin solo formulation are provided as supplementary information.

#### Knockdown and mortality results

**Fig. 3:**
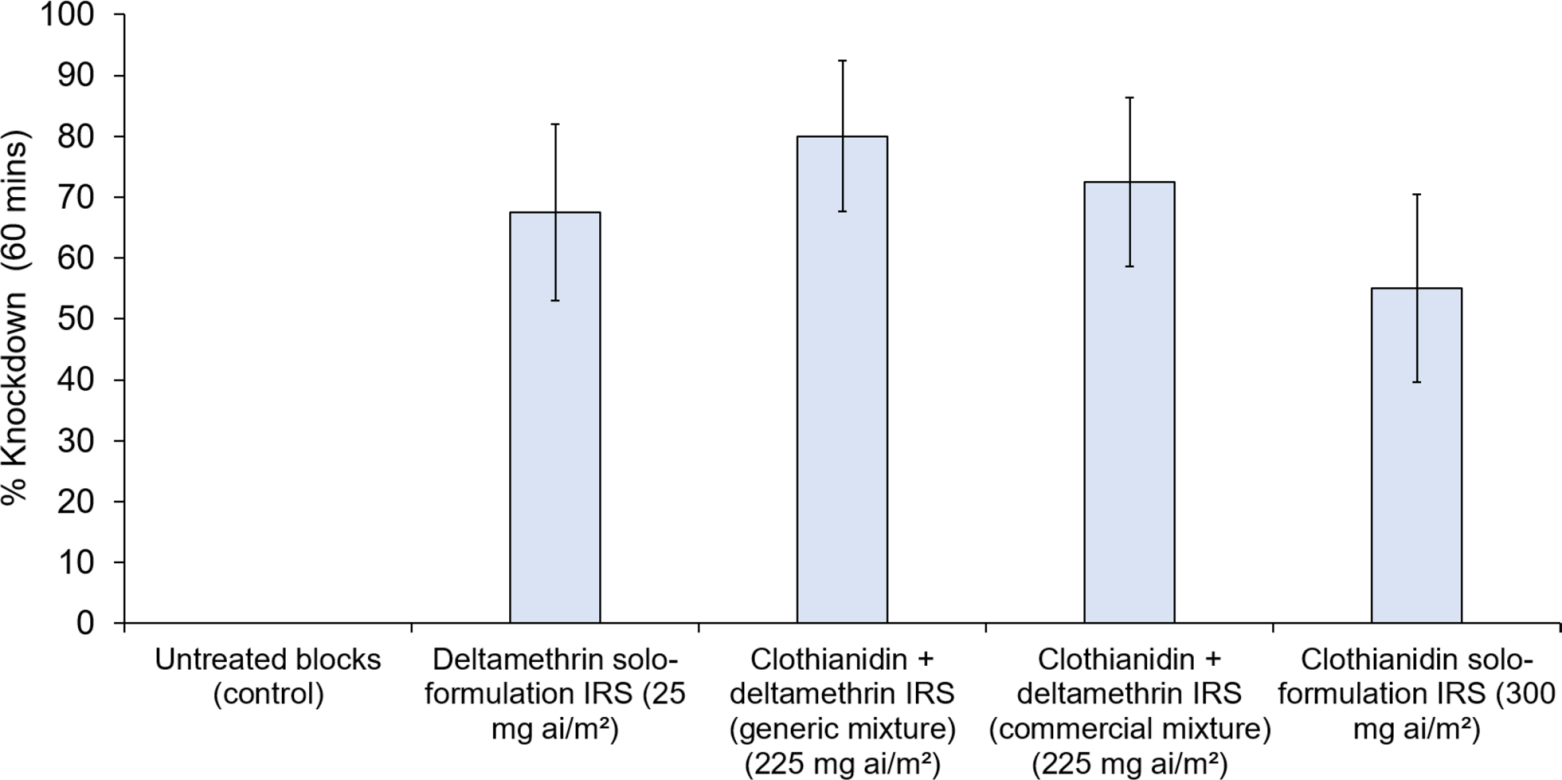
Knockdown after 60 min of the *Anopheles gambiae* sensu lato Covè strain in contact irritancy cone bioassays. A total of 40 mosquitoes were individually introduced into cones attached to treated cement blocks and exposed for 30 min. Error bars represent 95% confidence intervals

**Fig. 4:**
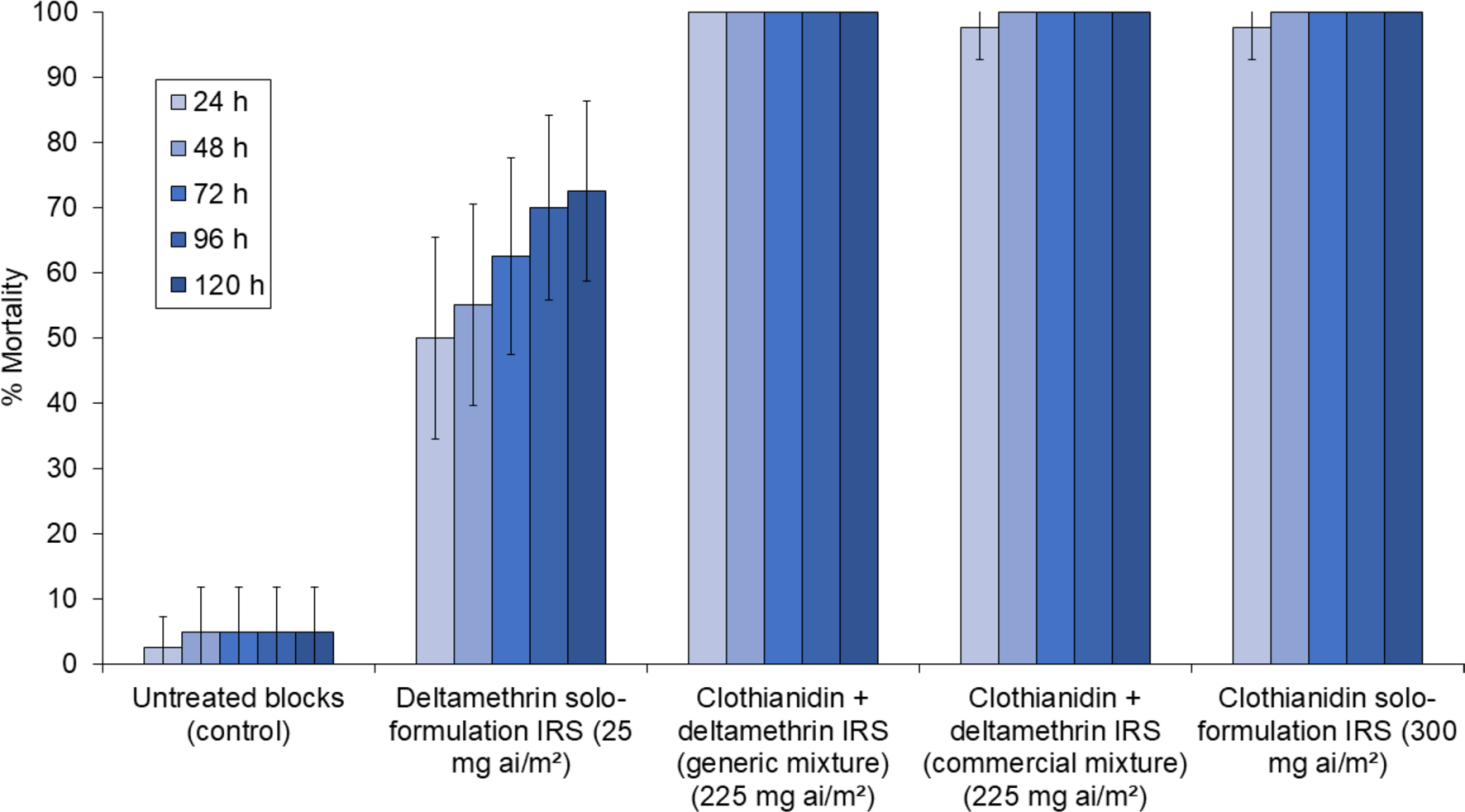
Mortality every 24 h up to 120 h of the *Anopheles gambiae* sensu lato Covè strain in contact irritancy cone bioassays. A total of 40 mosquitoes were individually introduced into cones attached to treated cement blocks and exposed for 30 min. Error bars represent 95% confidence intervals

Knockdown after 60 min of the pyrethroid-resistant Covè strain was lower with clothianidin solo formulation IRS (55%) compared to the generic clothianidin-deltamethrin mixture (80%), the commercial clothianidin-deltamethrin mixture (73%), and the deltamethrin-only (68%) treatments (Fig. 3). The lowest overall mortality rate after 120 h was achieved with the deltamethrin-only IRS (73%). In contrast, the clothianidin solo formulation and clothianidin-deltamethrin mixtures induced 100% mortality after 120 h (Fig. 4). While all clothianidin-based IRS killed ≥ 98% of mosquitoes within 24 h of exposure, mortality with the deltamethrin-only formulation was 50% at 24 h and increased to 73% at 120 h. Knockdown was 0% with the untreated control blocks while mortality was < 5%.

## Discussion

This laboratory study compared the contact irritancy of an IRS formulation containing clothianidin alone (SumiShield® 50WG) to clothianidin-deltamethrin mixture formulations against pyrethroid-resistant *An. gambiae* s.l. from Benin using a modified version of the WHO cone bioassay. We hypothesised that the clothianidin-deltamethrin mixture would elicit superior contact irritancy to the clothianidin solo formulation due to the irritant properties of its pyrethroid component. Our findings support this hypothesis showing a significantly enhanced contact irritancy response in mosquitoes exposed to the clothianidin-deltamethrin mixture relative to the clothianidin solo formulation.

In the video cone bioassays, the IRS formulation containing clothianidin alone induced no significant contact irritancy against pyrethroid-resistant *An. gambiae* s.l. relative to the untreated control. Although no published trials have evaluated the contact irritancy of clothianidin against mosquito vectors, this finding is consistent with several studies on agricultural pests showing that neonicotinoids elicit little to no irritancy or repellency [20–22]. Contact irritancy observed with the clothianidin-deltamethrin mixtures was therefore attributable to the irritant and excitorepellent properties of pyrethroids—a known characteristic of this insecticide [23]. The similarity in contact irritancy responses between the clothianidin-deltamethrin WP mixtures and the deltamethrin WG confirmed that the enhanced irritancy was due to the pyrethroid and was not associated with formulation differences.

Despite the increased contact irritancy of the clothianidin-deltamethrin mixture compared to the clothianidin solo formulation, both insecticides induced 100% mortality of pyrethroid-resistant *An. gambiae*, which was higher compared to the deltamethrin IRS formulation. The additional mortality with the clothianidin-based IRS can be attributed to the continued susceptibility of the Covè strain to neonicotinoids as demonstrated in the susceptibility bioassays. While both insecticides show potential to improve control of pyrethroid-resistant malaria vectors, the enhanced contact irritancy of an IRS insecticide formulation may affect its capacity to kill malaria vectors in a field setting compared to a less irritant formulation by reducing contact time between vectors and insecticide-treated surfaces. Previous experimental hut trials in Benin which evaluated the efficacy of IRS with the pyrrole insecticide chlorfenapyr either alone or as a mixture with the pyrethroid alpha-cypermethrin demonstrated reduced levels of mosquito mortality with the mixture compared to the solo formulation (43% vs. 63% [16] and 18–22% vs. 38–46% [24]). Similar findings were reported in a trial comparing the efficacy of IRS with a clothianidin solo formulation to a clothianidin-deltamethrin mixture, although it was argued that the difference in mortality was modest (70–71% vs. 72–78%) compared to the studies with chlorfenapyr IRS and may not have any operational significance [11].

In addition to killing mosquitoes, malaria transmission control can be enhanced by disrupting human-vector contact [25], which may suggest some potential benefit of the irritant effects of the pyrethroid in the IRS mixture through reducing mosquito house entry and increasing early exiting. However, because of the 10–14 day extrinsic incubation period of the malaria parasite [26], modelling studies predict that mass vectorial killing will prevent more malaria than the personal protection acquired from sleeping in a sprayed house [27]. For this reason, WHO prioritises IRS products providing high levels of vector mortality and community protection, with reductions in human-vector contact through increased exophily and deterrence considered a secondary benefit [28]. Despite this, we did not observe higher mortality with the clothianidin solo formulation we tested in this study (SumiShield® 50WG) compared to the clothianidin-deltamethrin mixture. It is therefore unclear whether the enhanced irritancy of the commercial mixture formulation will translate into a reduction in its capacity to kill malaria vectors compared to the solo formulation we tested. Field studies directly comparing the efficacy of these formulation types are warranted to establish the impact of this enhanced contact irritancy on the performance of WHO-prequalified clothianidin IRS mixture formulations containing pyrethroids.

One of the rationales supporting the co-formulation of clothianidin and deltamethrin into an IRS mixture was to provide opportunity for insecticide resistance management (IRM). Deploying mixtures of insecticides with different modes of action is expected to reduce selection for insecticide resistance because vectors surviving exposure to one component of the mixture due to the resistance are likely to be killed by the other [29]. Recent modelling studies on malaria vectors [30–32] have suggested that mixtures of pyrethroids and non-pyrethroid insecticides may reduce selection for resistance compared to use of the constituent components alone. The success of mixtures may, however, rely on the target vector population remaining susceptible to both compounds in the mixture [33]. Given that pyrethroid resistance is pervasive in malaria vector populations throughout sub-Saharan Africa, the capacity of insecticide mixtures containing pyrethroids to delay selection of resistance to the partner insecticide is unclear. Further empirical field studies are needed to establish the IRM potential of insecticide mixtures containing pyrethroids in the context of high pyrethroid resistance and the increasing use of clothianidin-deltamethrin IRS mixtures and dual-active ingredient insecticide-treated nets for malaria control.

## Conclusions

This study provides evidence of enhanced contact irritancy of the pyrethroid in clothianidin-deltamethrin IRS mixtures resulting in shorter mosquito contact times with treated blocks compared to the solo-clothianidin formulation. Despite this, the two product types performed similarly under laboratory conditions and it is unclear how the enhanced contact irritancy of the mixture will affect the comparative performance of available WHO-prequalified formulation types when deployed at community scale. Further trials are needed to directly compare their efficacy under field conditions and establish the impact of this enhanced contact irritancy on the performance of IRS mixture formulations containing pyrethroids.

### Supplementary Information

**Additional file 1: Figure S1.** Experimental set-up for contact irritancy cone bioassays with video camera set-up in view of the cones. **Table S1.** Summary contact irritancy bioassay results with the pyrethroid-resistant *Anopheles gambiae* sensu lato Covè strain. A total of 40 mosquitoes were individually introduced into cones attached to treated cement blocks and filmed for 3 min to record take-offs. *Values in the same row sharing a superscript letter do not differ significantly at the 5% level (i.e. *p* > 0.05) according to regression analysis.

## Data Availability

The aggregated datasets used and/or analysed during the current study are provided as supplementary information.

## Abbreviations

cRCT: Cluster-randomised controlled trial
CREC: Centre de Recherche Entomologique de Cotonou
IRM: Insecticide resistance management
IRS: Indoor residual spraying
LSHTM: London School of Hygiene & Tropical Medicine
PBO: Piperonyl butoxide
P450: Cytochrome P450 monooxygenase
