# Supplementary material for: Laboratory evaluation of the contact irritancy of a clothianidin solo formulation vs. clothianidin-deltamethrin mixture formulations for indoor residual spraying against pyrethroid-resistant Anopheles gambiae sensu lato
Source: Parasit Vectors. 2024 Apr 10;17:183. doi: 10.1186/s13071-024-06265-x (PMC11005223; doi:10.1186/s13071-024-06265-x)
Supplement: Supplementary file 1 — Additional file 1: Figure S1. Experimental set-up for contact irritancy cone bioassays with video camera set-up in view of the cones. Table S1. Summary contact irritancy bioassay results with the pyrethroid-resistant Anopheles gambiae sensu lato Covè strain. A total of 40 mosquitoes were individually introduced into cones attached to treated cement blocks and filmed for 3 min to record take-offs. *Values in the same row sharing a superscript letter do not differ significantly at the 5% level (i.e. p > 0.05) according to regression analysis. [file 13071_2024_6265_MOESM1_ESM.docx]

**Additional file 1**

**Figure S1:** Experimental set-up for contact irritancy cone bioassays with video camera set up in view of the cones.

Video links showing the irritating effects of the clothianidin-deltamethrin mixture compared to the absence of irritating effects with a clothianidin solo-formulation in contact irritancy cone bioassays:

<https://vimeo.com/920374984?share=copy>

<https://vimeo.com/927168781?share=copy>


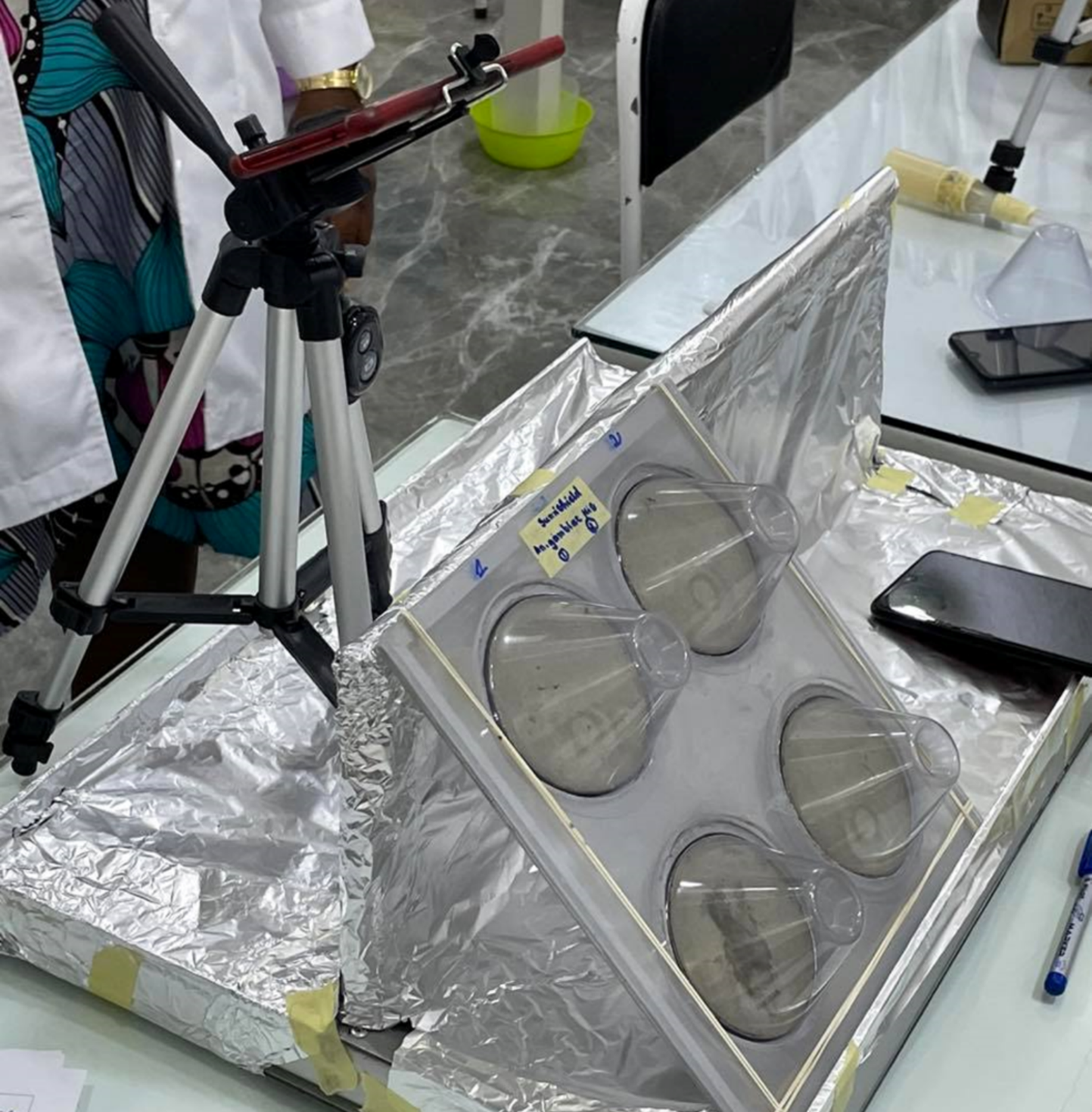


**Table S1:** Summary contact irritancy bioassay results with the pyrethroid-resistant *Anopheles gambiae sensu* lato Covè strain. *A total of 40 mosquitoes were individually introduced into cones attached to treated cement blocks and exposed for a total of 30 mins. *Values in the same row sharing a superscript letter do not differ significantly at the 5% level (i.e. p>0.05) according to binomial regression analysis.*

| **Treatment** | **Untreated blocks (control)** | **Deltamethrin-only** | **Clothianidin + Deltamethrin (generic)** | **Clothianidin + Deltamethrin (commercial)** | **Clothianidin-only** |
| --- | --- | --- | --- | --- | --- |
| **Application rate** | **̶** | **25 mg ai/m^2^** | **Total ai content: 225 mg ai/m^2^** | **Total ai content: 225 mg ai/m^2^** | **300 mg ai/m^2^** |
| **N exposed** | 40 | 40 | 40 | 40 | 40 |
| **N total take-offs** | 125 | 328 | 278 | 243 | 138 |
| **Mean take-offs/mosquito*** | 3.1^a^ | 8.2^b^ | 7.0^b^ | 6.1^b^ | 3.5^a^ |
| **95% CIs** | 2.3–3.9 | 6.4–10.0 | 5.8–8.1 | 4.6–7.6 | 2.7–4.2 |
| **N KD 60 mins** | 0 | 27 | 32 | 29 | 22 |
| **% KD 60 mins** | 0 | 67.5 | 80 | 72.5 | 55 |
| **95% CIs** | ̶ | 53.0–82.0 | 67.6–92.4 | 58.7–86.3 | 39.6–70.4 |
| **N dead 24 h** | 1 | 20 | 40 | 39 | 39 |
| **% dead 24 h** | 2.5 | 50 | 100 | 97.5 | 97.5 |
| **95% CIs** | 0–7.3 | 34.5–65.5 | ̶ | 92.7–100 | 92.7–100 |
| **% corrected dead 24 h** | ̶ | 48.7 | 100 | 97.4 | 97.4 |
| **N dead 48 h** | 2 | 22 | 40 | 40 | 40 |
| **% dead 48 h** | 5 | 55 | 100 | 100 | 100 |
| **95% CIs** | 0–11.8 | 39.6–70.4 | ̶ | ̶ | ̶ |
| **% corrected dead 48 h** | ̶ | 52.6 | 100 | 100 | 100 |
| **N dead 72 h** | 2 | 25 | 40 | 40 | 40 |
| **% dead 72 h** | 5 | 62.5 | 100 | 100 | 100 |
| **95% CIs** | 0–11.8 | 47.5–77.5 | ̶ | ̶ | ̶ |
| **% corrected dead 72 h** | ̶ | 60.5 | 100 | 100 | 100 |
| **N dead 96 h** | 2 | 28 | 40 | 40 | 40 |
| **% dead 96 h** | 5 | 70 | 100 | 100 | 100 |
| **95% CIs** | 0–11.8 | 55.8–84.2 | ̶ | ̶ | ̶ |
| **% corrected dead 96 h** | ̶ | 68.4 | 100 | 100 | 100 |
| **N dead 120 h** | 2 | 29 | 40 | 40 | 40 |
| **% dead 120 h** | 5 | 72.5 | 100 | 100 | 100 |
| **95% CIs** | 0–11.8 | 58.7–86.3 | ̶ | ̶ | ̶ |
| **% corrected dead 120 h** | ̶ | 71.1 | 100 | 100 | 100 |
